# New terbium complex as a luminescent probe for determination of chlorogenic acid in green coffee and roasted coffee infusions

**DOI:** 10.1007/s00216-022-04411-x

**Published:** 2022-11-15

**Authors:** Alla Yegorova, Yuliia Skrypynets, Inna Leonenko, Axel Duerkop

**Affiliations:** 1 A. V. Bogatsky Physico-Chemical Institute, National Academy of Sciences of the Ukraine, Lustdorfskaya Doroga 86, Odessa, 65080 Ukraine; 2 Institute of Analytical Chemistry, Chemo- and Biosensors, University of Regensburg, Universitätsstrasse 31, 93040 Regensburg, Germany

**Keywords:** Chlorogenic acid, Luminescence, Probe, Terbium complex, Quenching

## Abstract

Green coffee is coming into vogue as a food that contains remarkable contents of antioxidants like chlorogenic acid (ChA) and induces mild stimulation to the consumer. While most methods for determination of ChA require chromatographic separation prior its quantitation, we present the first probe and a simple, sensitive and validated luminescence method for the determination of chlorogenic acid in green and roasted coffee infusion samples that does not require a chromatographic separation. ChA can remarkably quench the luminescence intensity of the Tb^3+^ complex with 1-(furan-2-ylmethyl)-4-hydroxy-*N*-(4-methylpyridin-2-yl)-2-oxo-1,2,5,6,7,8-hexahydroquinoline-3-carboxamide (R_3_) in aqueous solution containing urotropine buffer at a near neutral pH 7.5 at λ_exc_ = 315 nm and λ_em_ = 545 nm. Under optimal conditions, the quenching of the luminescence intensity is directly proportional to the concentration of ChA in the range of 0.5–30 μg/mL, and the detection limit is 180 ng/mL. From measurements of luminescence decay time, it was determined that both static and dynamic quenching is induced upon coordination of ChA to Tb-R_3_. The related quenching constants are K_S_ = 5.97∙10^4^ M^−1^ and K_D_ = 1.05⋅10^4^ M^−1^. Finally, the method was applied successfully to the determination of ChA in real green and roasted coffee infusion samples and validated by HPLC to yield very closely matching concentrations of both methods. Therefore, this method can serve for a simple quality control of total ChA contents in coffee prior and after roasting.

**Graphical Abstract:**

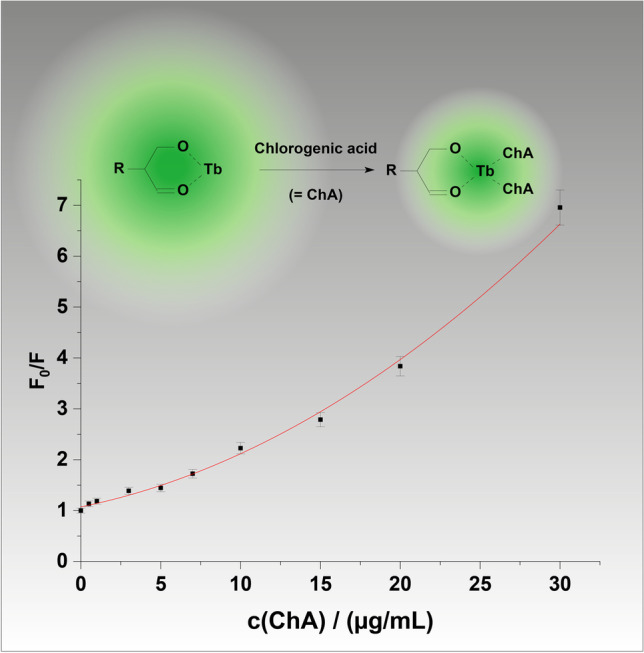

**Supplementary Information:**

The online version contains supplementary material available at 10.1007/s00216-022-04411-x.

## Introduction

**Scheme 1.**
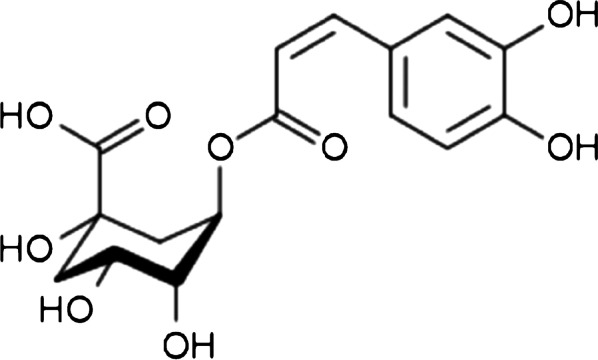
Structure of chlorogenic acid (ChA)

Chlorogenic acid (ChA)—3-(3,4-dihydroxycinnamoyl)quinic acid (see Scheme 1)—is one of the main substances contained in, e.g. coffee beans or blueberry leaves, and can be chemically regarded as an esterification product between quinic acid and caffeic acid. Being a polyphenol, it shows remarkable antioxidant, anti-carcinogenic, anti-inflammatory therapeutic and analgesic action [1, 2]. The content of chlorogenic acid in coffee depends on the growing area, soil, climatic, ripeness state of the coffee beans and storage conditions, etc. [1]. The properties of green coffee are still being studied, as are its effects on human health. It is a product that is especially recommended and known for its high content of chlorogenic acid. It contains about 5–12 g of chlorogenic acid per 100 g of grain [3]. Green coffee extract is mostly available as a nutraceutical in capsules but also as a supplement in beverages, chocolate and chewing gum. Most often, the total polyphenolic components, including chlorogenic acid, are determined by high-performance liquid chromatography (HPLC or UHPLC) coupled with photometric detection [2–6].

Few methods have been reported for the determination of chlorogenic acid including HPTLC [7, 8], spectrophotometric [9–11] and HPLC-spectrofluorometric [12] methods. While HPTLC provides high sensitivity, yet specialized equipment, photometry is simple, but displays too little sensitivity. HPLC provides superb separation and identification capability, especially when coupled with MS detection. However, HPLC instruments are quite expensive to purchase and require regular maintenance, whereas luminescence provides a similarly sensitive detection with less instrumental demand. If luminescence is combined with an appropriate probe that shows high binding constants to its target and high brightness, trace analysis is achieved even with inexpensive instruments. Therefore, new probes and luminescent methods could represent a promising avenue for quantitation of ChA.

The analytical application of lanthanide-sensitized luminescence has created great interest for a long time. The main advantages of lanthanide chelates as probes in luminescence spectrometry include large Stokes' shifts, narrow emission bands and long luminescence lifetimes. Moreover, their luminescence can be considerably increased by coordination of so-called antenna ligands with high molar absorbance and high quantum yield [13–15]. Such complexes are ideal probes for luminescence assays based on changes of luminescence intensity or lifetime. In the last years, the analytical use of sensitized lanthanide luminescence as well as its response with decrease or enhancement of luminescence intensity or lifetime towards the presence of relevant biomolecules demonstrated their potential as useful probes [16–18].

As chlorogenic acid is known for its complexation ability towards, e.g. Fe^3+^, we reasoned that ChA should also show coordination to lanthanide complexes with suitable stoichiometry and thereby have an impact on the luminescence properties of such a complex. The potential of those as luminescent probes for bioanalytical purposes has been recently described [17]. We then characterized three different new Tb-quinolone complexes as potential probes for chlorogenic acid due to their high molar absorbances, long lifetimes and high quantum yields. Furthermore, the complexation constants of ChA to the best Tb^3+^ probe and the stoichiometry upon coordination were determined. Importantly, we could derive a method for quantitation of ChA in real coffee samples based on the quenched emission of the Tb^3+^ ion, especially the hypersensitive ^5^D_4_ → ^7^F_5_ transition at 545 nm. It works at a near neutral pH of 7.5 and has a wide working range and a low LOD. The suggested method is rapid, simple, sensitive and can be used for the determination and quality control of ChA in green and roasted coffee as validated by HPLC with photometric detection.

## Materials and methods

### Instrumentation

All luminescence measurements (luminescence spectra, excitation spectra and lifetimes) were carried out on a Cary Eclipse (Varian, Australia) luminescence spectrophotometer in the range (220–700 nm) equipped with a xenon flash lamp in a 1.00 cm quartz cell. The excitation and emission monochromator band widths were 5 nm. The excitation wavelength was set at 315 nm, and the luminescence was measured using the peak height at 545 nm. All measurements were performed at room temperature (21–23 °C) which should be kept at this level. Absorption spectra were recorded with a UV-2401 PC (Shimadzu, Japan) spectrophotometer.

A pH meter (Lab 850, Schott Instruments GmbH, Germany) was used for pH adjustment. HPLC chromatograms were obtained using an Agilent Technologies 1200 Series chromatograph with isocratic elution under the conditions given in the “Preparation of the lanthanide complex” section. Peak areas were automatically integrated by the Agilent software.

### Reagents

All of the used chemicals were of analytical grade or chemically pure and doubly distilled water was used, throughout. The standard solution of terbium (III) chloride (1.00∙10^–1^ mol/L) was prepared from a high purity oxide. The concentration of the metal was determined by complexometric titration with Arsenazo I as the indicator.

The ligands R_1_–R_3_ were synthesized as described in [19]. The stock standard solutions of the ligands (1.00∙10^–3^ mol/L) were prepared by dissolving accurate weights of the solid compounds in dimethylformamide. The stock standard solutions of the ligands were diluted to 1.00∙10^–4^ mol/L with water.

An accurately weighted standard sample of 50.0 mg of chlorogenic acid (from Acros, CAS 327–97-9) was dissolved in water, placed into a 50.0-mL volumetric flask, stirred and diluted to the mark with water. Herewith, a standard solution of 1.00 mg/mL was obtained. The stock standard solution of ChA was diluted to 100 μg/mL with water before being used. Similarly, standard samples of caffeine (from Sigma-Aldrich, CAS 58–05-2) and caffeic acid (from Sigma-Aldrich, CAS 331–39-5) were prepared. A green coffee sample “Gregincof” (Vietnam robusta) was supplied by Buon Ma Thuot coffee company (Vietnam). A roasted coffee sample “Cerrado” (BRAZIL, arabica) was supplied by Kyiv Roasting Company (Ukraine).

An 2.85 mol/L (40%) urotropine buffer was prepared by dissolving 40.0 g of urotropine in a 100-mL volumetric flask with water and adjusting the pH to 7.5 with hydrochloric acid and then diluting with water to 100 mL. Ammonium acetate buffers of various pH were prepared to determine the pH-dependence of the luminescence emission of the Tb-R complexes.

### Preparation of the lanthanide complex

The complex of a ligand with Tb^3+^ ions was prepared by mixing the respective ligand at a concentration of 1.00∙10^−5^ mol/L and Tb^3+^ with 1.00∙10^−5^ mol/L in a molar ratio of 1:1 in water at room temperature. The Tb-R_3_ complex is water soluble up to a concentration of 1.00∙10^−3^ mol/L as no precipitation was observed.

### Luminescence spectra of Tb-R_3_ complex in presence of different concentrations of ChA

0; 0.050; 0.100; 0.300; 0.500; 0.700; 1.00; 1.50; 2.00; 3.00 mL of ChA working solution (100 µg/mL), respectively, were added into volumetric flasks. 1.00 mL of a working terbium chloride solution (1.00∙10^−4^ mol/L), 1.00 mL of R_3_ working solution (1.00∙10^−4^ mol/L) and 0.500 mL of urotropine buffer (40 %) were added to each of these volumetric flasks. The solutions were diluted with water to 10.0 mL and stirred. The luminescence intensity (F) was measured at λ_exc_/λ_em_ = 315/545 nm after 5 min. Concentrations of the samples were derived from the related calibration curve.

### Luminescence determination of ChA in green coffee samples

The green coffee samples were prepared for HPLC and luminescent analysis based on the following procedure according to a published method [20]. 100 mg of ground green coffee sample were accurately weighed in 25 mL beakers, 10.0 mL of distilled water were added to each sample, and the samples were boiled for 30 min while stirring. Then, the coffee samples were cooled and the solution was filtered through a 0.45-μm filter. The clear filtrate was used for analysis and diluted as required. 0.300 mL of the filtrate were placed into a 10.0-mL volumetric flask. Further, 1.00 mL of a working terbium chloride solution (1.00∙10^−4^ mol/L), 1.00 mL of R_3_ working solution (1.00∙10^−4^ mol/L) and 0.500 mL of urotropine buffer were added to each of these volumetric flasks. Then, water was added to make the volume up to 10.0 mL, and luminescence intensity was measured at λ_exc_/λ_em_ = 315/545 nm. The luminescence intensity of the control solution (F_0_) which contains all components with the exception of ChA was recorded at the same time.

### HPLC determination of ChA in green coffee

The HPLC conditions were as follows: column, reverse phase – ODS 150 × 4.6 mm, flow rate 0.6 mL/min, column temperature 40 °C, photometric detection wavelength 280 nm, mobile phase methanol:5.00 mM KH_2_PO_4_ (30/70), sample volume 10 μL. Note that the number of theoretical plates should not be less than 2000 as calculated based on the chlorogenic acid peak. The area under the peak was used for calibration.

## Results and discussion

### Spectral characteristics of the ligands and their Tb complexes

The absorption spectra of the ligands in aqueous solutions show two bands in the UV with peak wavelengths around 240 and 310 nm. The molar absorption coefficients (22,800–25,300 L∙mol^−1^∙cm^−1^) of these bands are high, and therefore, the ligands provide efficient absorption of excitation light. The triplet energy levels (E) of the ligands were calculated from phosphorescence spectra of the respective Gd complexes at 77 K (Table 1). This energy is higher than the energy of the level of the first excited Tb^3+^ ion state (^5^D_4_; 20,500 cm^−1^) which enables an energy transfer from any ligand R to the lanthanide ion.

**Table 1:**
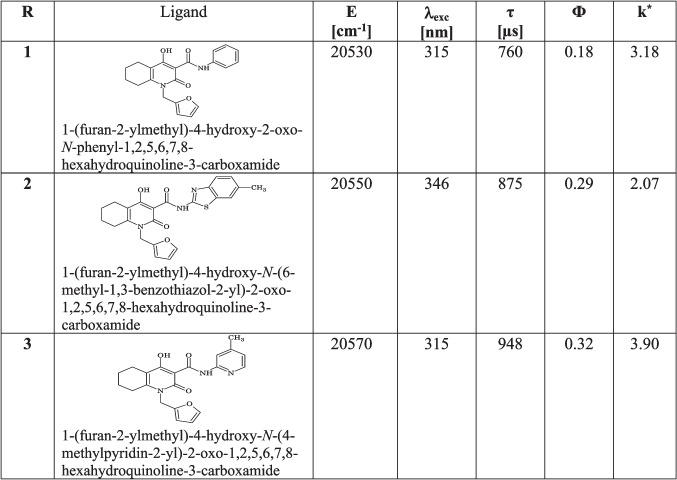
Triplet-state energy levels (*E*) of ligands R_1_–R_3_; excitation maxima (*λ*_exc_), lifetimes (τ) and quantum yields (*Φ*) of the Tb complexes with these ligands (c_Tb_^3+ ^= c_R_ = 1∙10^−5^ mol/L; pH 7.5; λ_em_ = 545 nm) and luminescence quenching factors of Tb-R complexes in the presence of ChA

^*^Quenching ratio of the luminescence intensity of the terbium complex in absence of ChA to the luminescence intensity of the terbium complex in the presence of ChA (c_ChA_ = 20 µg/mL).

**Fig. 1:**
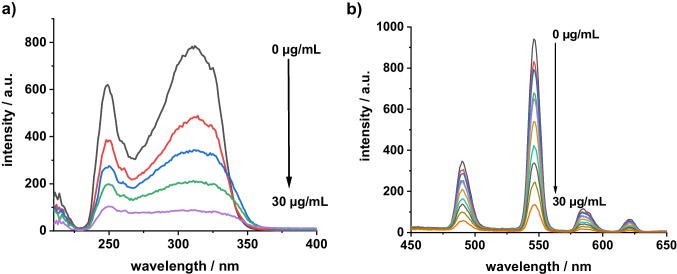
Excitation spectra of the Tb-R_3_ complex (**a**) and luminescence spectra of Tb-R_3_ in the presence of various concentrations of ChA (**b**) (c_Tb_^3+ ^= c_R3_ = 1.00∙10.^−5^ mol/L; λ_exc_/λ_em_ = 315 nm/545 nm)

Figure 1a shows the excitation spectrum of the Tb-R_3_ complex monitored at an emission wavelength of 545 nm. The excitation maximum is found at 315 nm for both, the Tb-R_1_ complex and the Tb-R_3_ complex, while the excitation maximum of the Tb-R_2_ complex is more longwave at 346 nm. The emission bands of the Tb-R_3_ complex (Fig. 1b) are located at 490, 545, 585 and 620 nm, obviously generated through the ^5^D_4_ → ^7^F_6_, ^5^D_4_ → ^7^F_5_, ^5^D_4_ → ^7^F_4_ and ^5^D_4_ →^7^F_3_ transitions, respectively. The other two Tb complexes with R_1_ and R_2_, respectively, show a very similar pattern of emission bands.

The quantum yields of the complexes with R_1_–R_3_ were determined in urotropine buffer of pH 7.5 relative to quinine sulphate at λ_exc_ = 315 nm. They are given in Table 1 and vary from 0.18 to 0.32 with Tb-R_3_ having the highest quantum yield. Moreover, Tb-R_3_ has the longest luminescence lifetime of almost 1 ms. The lifetimes of the other two complexes are also in a typical range for luminescent Tb-complexes. Organic solvents were found to decrease the luminescence intensity of Tb-R_3_ by 40–70% (see Fig. S1 in the supplementary information) as compared to pure water. The higher quantum yield and longer lifetimes suggested the use of complex Tb-R_3_ as the most suitable probe for the luminescent determination of ChA.

**Fig. 2:**
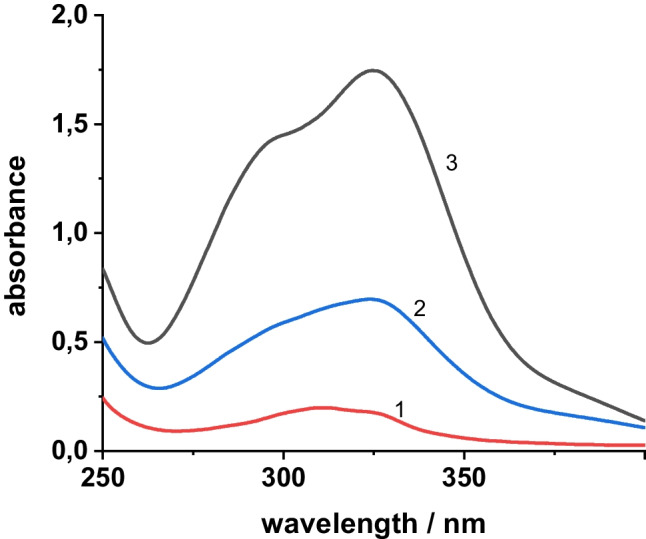
Absorption spectra of Tb-R_3_ (1), Tb-R_3_-ChA (2), ChA (3) (c_Tb_^3+ ^= c_R3_ = 1.00∙10.^−5^ mol/L, c_ChA_ = 10.0 µg/mL)

The luminescence spectra of the Tb-R_3_-ChA complex are similar to those of free Tb-R_3_, but the luminescence intensity is increasingly quenched in the presence of increasing amounts of ChA (Fig. 1b). In presence of 30 µg/mL of ChA, only 16% of the original intensity remains. On comparing the quenching ratio of all complexes (Table 1, last column), it is obvious that Tb-R_3_ is quenched most efficiently among all three complexes in presence of ChA. This additionally suggests the use of the Tb-R_3_ complex as a probe for ChA by means of luminescence quenching. Moreover, the interaction of the Tb-R_3_ complex with ChA causes a remarkable bathochromic shift of 20 nm of the absorption maximum from 310 to 330 nm (Fig. 2). This points to a strong coordination interaction between Tb-R_3_ with ChA.

### Effect of pH and of stoichiometry

**Fig. 3:**
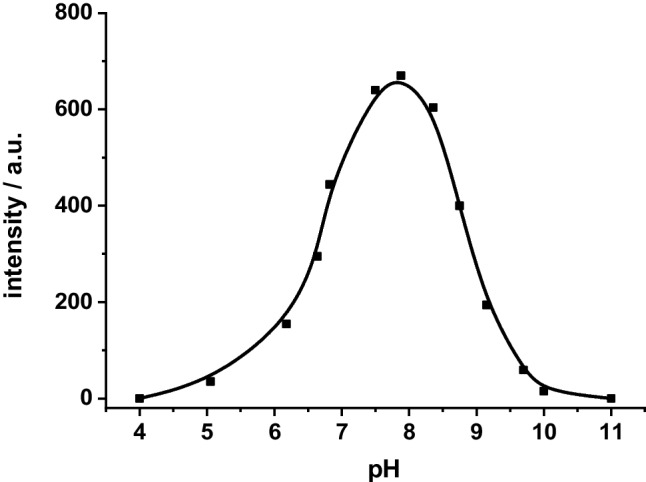
Effect of pH on the luminescence intensity of the complex Tb-R_3_ (c_Tb_^3+ ^= c_R3_ = 1.00∙10.^−5^ mol/L)

The complexation of Tb(III) with the ligands occurs in a wide range of pH values from 4 to 11 (Fig. 3). The highest luminescence intensity of the complex Tb–R_3_ is observed at a neutral pH range of pH 7.0–8.5 at constant concentration of 1.00∙10^−5^ mol/L. Therefore, the pH of the solutions was maintained at 7.5 with urotropine buffer for further measurements.

Applying the restricted-logarithm method to the luminescence data, it was found that in case of molar ratios of Tb:R_3_ of until 1:1 or at equimolar ratio of Tb:R_3_, a complex with 1:1 stoichiometry and a lifetime (τ) of 948 µs is formed. If the ligand is present in excess, however, terbium forms a complex with R_3_ in a 1:3 molar ratio. This is further supported by the much longer lifetime of 1130 µs that is detected for the Tb-(R_3_)_3_ complex. Hence, to permit the coordination of ChA in a quenching assay, equal concentrations (1.00∙10^−5^ mol/L) of Tb^3+^ and R_3_ were regarded as optimal to form the luminescent probe and those were chosen for the further analytical experiments. When we then experimentally changed the concentration of ligand R_3_ at constant concentrations of Tb^3+^ (1.00∙10^−5^ mol/L) and ChA (20 μg/mL), we also found that a concentration of 1.00∙10^−5^ mol/L of both Tb^3+^and R_3_ is the optimum.

### Analytical performance of Tb-R_3_ for quantitation of chlorogenic acid

**Fig. 4:**
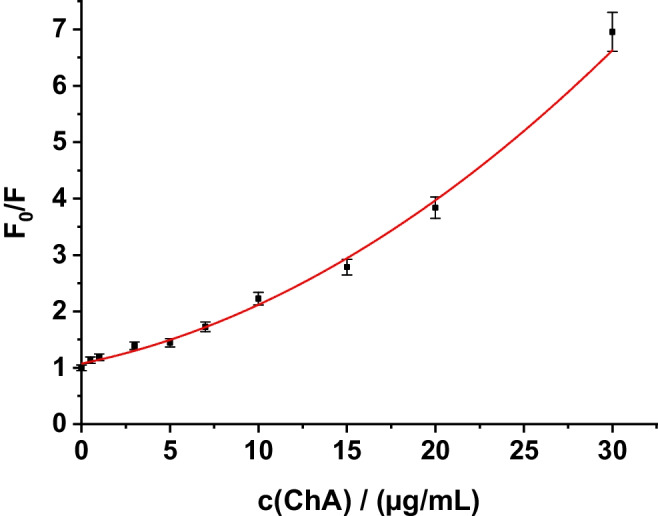
Non-linear Stern–Volmer plot for the determination of ChA (c _Tb_^3+ ^= c_R3_ = 1.00∙10^−5^ mol/L; λ_exc_/λ_em_ = 315/545 nm) (*n* = 3)

The strong luminescence quenching due to the presence of ChA pointed to set up a calibration plot that is based on a Stern–Volmer plot. Hence, the luminescence quenching of Tb-R_3_ in 1:1 molar ratio was determined at various concentrations of the quencher (ChA) and validated in terms of linearity, accuracy,—and intra-day precision and specificity. F_0_ and F were measured at λ_exc_ = 315 nm and λ_em_ = 545 nm, respectively, and a nonlinear Stern–Volmer plot was obtained (Fig. 4). The following equation was obtained as best fit: F_0_/F = 1.126 – 0.042c_ChA_ + 0.005c_ChA_^2^ with a very good correlation coefficient of 0.9962. Here, F_0_ and F are the relative luminescence intensities determined without and with ChA, respectively, and c_ChA_ is the concentration of chlorogenic acid in µg/mL. As can be seen from Fig. 4, the Stern–Volmer plot has an upward curvature and obeys the polynominal equation. This points to the presence of both, static and dynamic quenching. More detailed data on the quenching mechanism, lifetime data and quenching constants are given in the “Determination of luminescence quenching mechanism” section. The quenching of luminescence intensity is proportional to the concentration of chlorogenic acid in the range of 0.5–30 μg/mL, and the detection limit is 180 ng/mL (Table 2).

**Table 2:** Figures of merit of the luminescence determination of ChA with Tb-R_3_ according to the non-linear Stern–Volmer plot

Parameter	Result
Quantitation range [µg/mL]	0.5–30.0
LOD [µg/mL]	0.18
Correlation coefficient (*r*)	0.9962
Accuracy (*n* = 5) [%]	100.6
Precision Inter-day (*n* = 6) [%] Intra-day (*n* = 6) [%]	2.4 2.8
Specificity	specific

The precision of the method was established by acquiring the luminescence quenching at a ChA concentration of 10 µg/mL. For a series of 12 measurements, the relative standard deviation was 2.4% for the intra-day and 2.8% for the inter-day analysis, respectively (*P* = 95% confidence level) for ChA indicating high precision.

Accuracy of the method was evaluated by carrying out a recovery study at three different concentration levels of ChA. The results of the recovery study indicate that the proposed method is very accurate for the estimation of ChA in green coffee (Table 3). The recovery shows no systematic trend towards lower or higher levels of ChA.

**Table 3:** Recovery of ChA in solution (*n* = 5, *P* = 95%)

Amount added [µg/mL]	Amount found [µg/mL]	Recovery [%]	RSD [%]
3	3.05 ± 0.06	101.7	1.6
10	10.09 ± 0.15	100.9	1.2
20	19.85 ± 0.27	99.3	1.1
		Average recovery: 100.6	

The interference of caffeine and caffeic acid which are contained in higher concentrations in green coffee [20, 21] was studied by addition of these compounds to a solution of 10 μg/mL of ChA. Then, this mixture was added to a solution of the Tb-R_3_ complex, and the change of the luminescence intensity (ΔF) was determined as compared to a solution of ChA and Tb-R_3_ with the same concentrations, but without the interferent being present. As shown in Table 4, caffeine and caffeic acid had a very little effect on the luminescent determination of ChA. Hence, a high specificity is achieved by the proposed method. This suggests that the Tb-R_3_ complex is a reliable luminescent probe to determine ChA in green coffee**.**

**Table 4:** Tolerance limits of various interferents on the determination of 10 μg/mL of ChA

Interferent	Interferent-to-analyte ratio	ΔI [%]
Caffeine	2:1	− 0.05
Caffeic acid	2:1	− 1.5

**Fig. 5:**
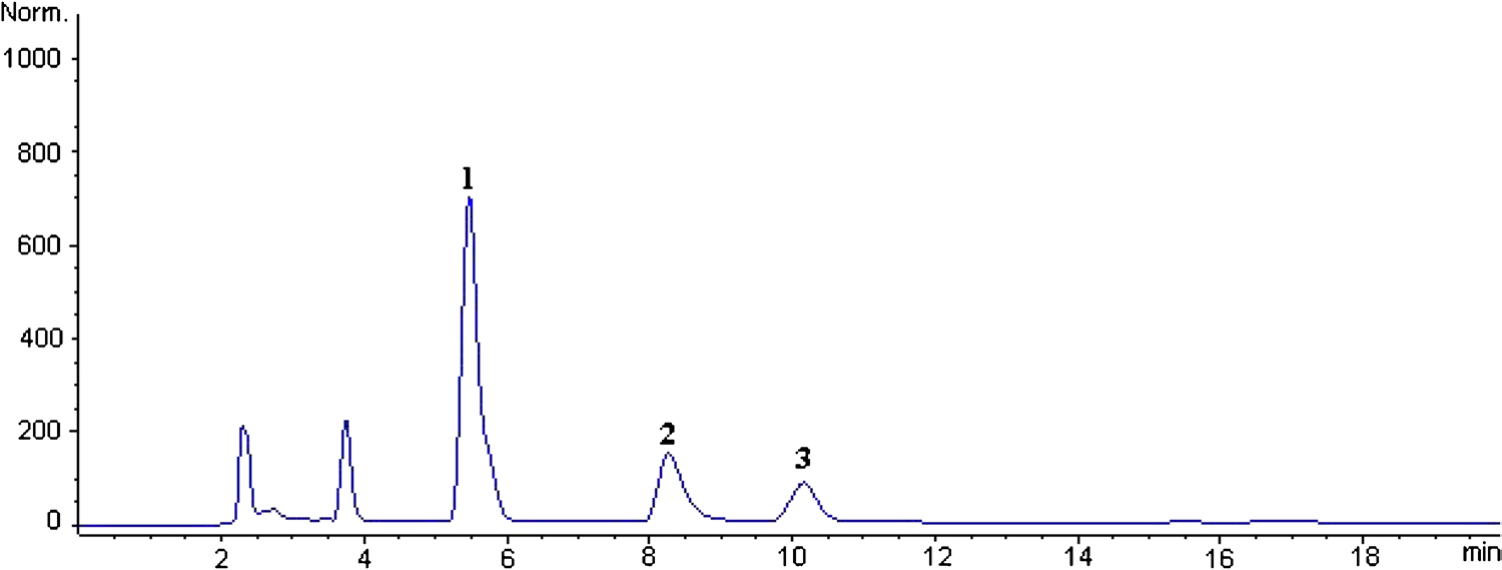
Chromatogram of green coffee sample: ChA (1); caffeine (2); caffeic acid (3)

Next, ChA was determined in real green and roasted coffee samples by luminescence quenching of Tb-R_3_ and validated with HPLC with photometric detection as described in the “HPLC determination of ChA in green coffee” section. A representative HPLC chromatogram is displayed in Fig. 5 and shows ChA eluting at the highest absorption peak at a retention time of 5.50 min. Additionally, caffeine and caffeic acid are found in typical concentrations [3, 20, 21] of 1.4 mg/100 mg and 0,85 mg/100 mg, respectively, in the green coffee sample. 1.2 mg/100 mg of caffeine and 0.65 mg/100 mg of caffeic acid were found in the roasted coffee sample. A comparison of the related concentrations of ChA in mg/100 mg (i.e. in %) found in the coffee samples by luminescence quenching and HPLC is shown in Table 5. The concentrations of ChA obtained match very well within the range of errors of the individual methods. Importantly, the standard deviations are very low, even though the number of samples (*n* = 5) was not very high. This demonstrates that both methods work precisely and that the determination of ChA with Tb-R_3_ also works reliably in real coffee samples. Additionally, the very similar contents of ChA found by both methods in real samples points to that Tb-R_3_ seem to deliver the total content of all ChA derivatives found in coffee [20, 21]. This is plausible because the luminescence response always occurs via coordination of the ChA derivatives with the free carboxylic acid of the quinic acid part of ChA, independently which coffee acid is esterified with the quinic acid part.

**Table 5:** Determination of ChA in coffee (*n* = 5, *P* = 95%)

	Sample №	ChA [mg/100 mg]					
		HPLC			Luminescence		
		Found	X_av_ ± ΔX	RSD [%]	Found	X_av_ ± ΔX	RSD [%]
Green	1 2 3 4 5	6.62 6.47 6.71 6.57 6.60	6.59 ± 0.18	2.25	6.70 6.58 6.74 6.81 6.63	6.73 ± 0.27	3.25
Roasted	1 2 3 4 5	0.74 0.73 0.71 0.73 0.69	0.72 ± 0.02	2.78	0.82 0.75 0.84 0.76 0.78	0.79 ± 0.05	4.90

Another welcome aspect is that the luminescence method not only permits the determination of the higher concentrations of ChA but also the much lower concentrations of ChA that remain after roasting of the coffee. This permits the quality control of the content of ChA in both green and roasted real coffee samples (i.e. prior and past roasting) with the very same method and hence makes the luminescence quenching method more valuable.

Compared to known methods for determination of ChA (Table 6), the HPLC methods offer better detection limits than our luminescence method. However, HPLC is associated with purchasing more expensive analytical instrumentation that is more complicated to operate. Luminescence equipment requires less than a quarter of the costs of an HPLC system, and the sample preparation is rapid and easy. Compared to spectrophotometric methods, our luminescence method is more sensitive, provides a wider dynamic range and covers the concentration range of ChA without dilutions during sample preparation.

**Table 6:** Overview on common methods for determination of chlorogenic acid in coffee samples

Method	λ[nm]	Linear range [µg/ml]	LOD [µg/ml]	Ref
HPLC–UV	325	0.07–12	-	3
HPLC–UV	326	0.05–1.25	0.013	4
MEKC-UV	325	25–900	0.98	5
HPLC–UV	330	9.69–1299	1.25	6
HPTLC-UV	366	20–300^*^	-	7
HPTLC-UV	330	12–240^*^	-	8
UV spectroscopy	324	48–176	16	9
NIR-vis spectroscopy	790	10–800	3.5	10
UV spectroscopy	330	0.5–5.0	0,2	11
Luminescence	545 (λ_em_)	0.5–30.0	0.18	This work

*- ng/band.

### Determination of luminescence quenching mechanism

Luminescence quenching experiments were carried out to explore the quenching mechanism and to determine quenching constants. Chlorogenic acid has a carboxyl functional group, which makes it amenable to coordinate to Tb^3+^. This group has a pK_a_ value of 3.55 [22] and is therefore deprotonated under the conditions (urotropine buffer of pH 7.5) used here. Hence, ChA can coordinate as a ligand that is countercharged with respect to Tb^3+^. The coordinative binding is confirmed by the strong red-shift of Tb-R_3_-ChA as compared to the absorption spectra of the free Tb^3+^-R_3_ complex and of ChA (Fig. 2). As a result, an energy loss of the Tb^3+^-R_3_ complex is observed which leads to the fluorescence quenching.

**Fig. 6:**
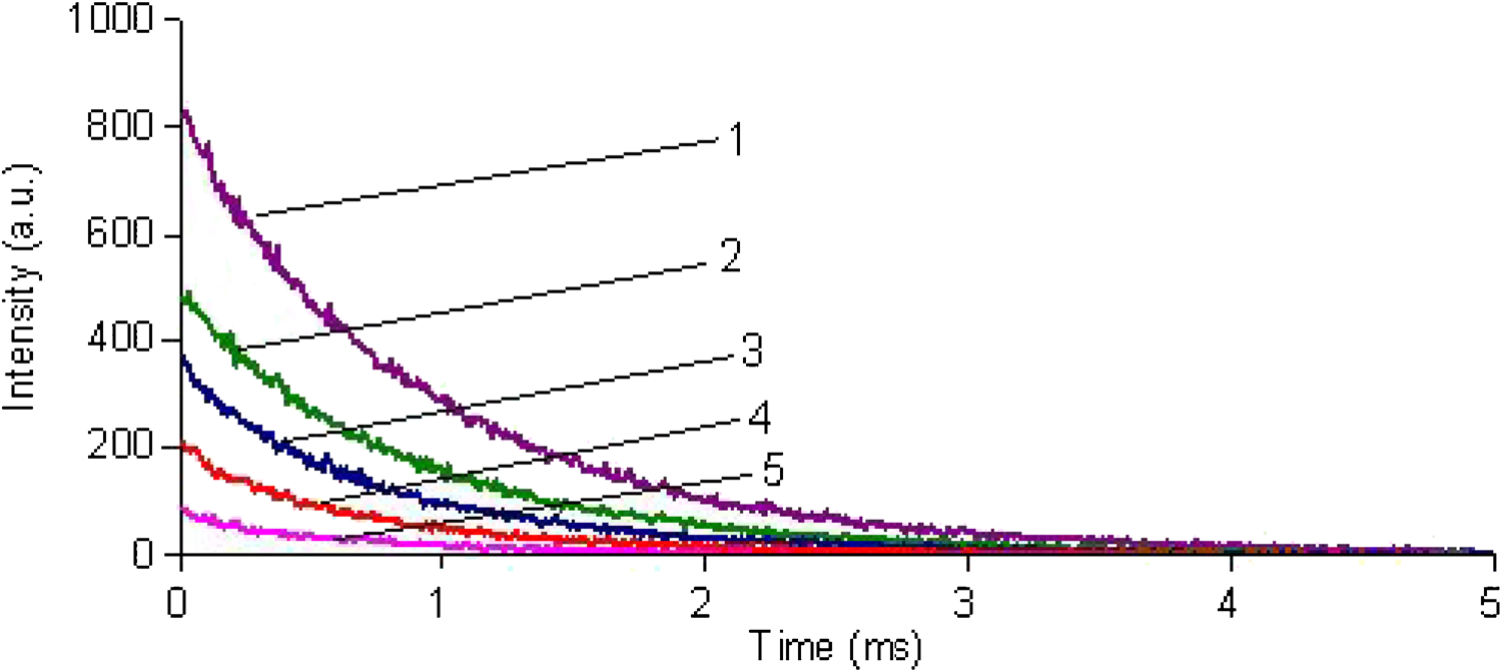
Luminescence decay curves of the Tb-R_3_ complex without (1) and in presence (2)–(5) of various concentrations of ChA (c _Tb_^3+ ^= c_R3_ = 1∙10.^−5^ mol/L; c_ChA_: (2) 1 µg/mL (τ = 880 µs); (3) 5 µg/mL (τ = 780 µs); (4) 10 µg/mL (τ = 725 µs); (5) 20 µg/mL (τ = 700 µs), λ_exc_/λ_em_ = 315 nm/545 nm)

The decay time τ of the terbium emission of the Tb-R_3_ complex is 948 µs. In the presence of increasing concentrations of ChA from 1 to 20 µg/mL, τ decreases from 880 to 700 µs (Fig. 6). This confirms a contribution of dynamic quenching to the overall quenching of Tb-R_3_.

From a Stern–Volmer plot of τ_0_/τ vs. c_ChA_, where τ_0_ is the average lifetime of the Tb-R_3_ complex, the slope of the linear function gives the dynamic quenching constant. The slope of the plot τ_0_/τ vs. c_ChA_ is consistent with a K_D_ of 1.05 ⋅ 10^4^ M^−1^. This coincides very well with the concentration of the quencher of 1.0∙10^−5^ mol/L where 50% of the intensity is quenched and which should also equal K_D_ [23].

We then calculated K_s_ based on the luminescence intensity of the plot shown in Fig. 4. This plot was converted to a plot of F_0_/F vs. c_ChA_/(mol/L). Accordingly, one can calculate the dynamic and static quenching constants using F_0_/F = 1 + B_1_⋅ c_ChA_ + B_2_ ⋅c_ChA_^2^ [23].

B_1_ of this plot equals K_D_ + K_S_ and B_2_ equals K_D_⋅K_S_, where K_D_ is the dynamic quenching constant and K_S_ is the static quenching constant. This equals F_0_/F = 1 + (K_D_ + K_S_)⋅ c_ChA_ + K_D_⋅K_S_ ⋅ _ChA_^2^. In the fit function, we calculated F_0_/F = 1.120 + 14,985⋅ c_ChA_ + 6.265 ⋅ _ChA_^2^ which results in K_D_ + K_S_ = 14,985 M^−1^ and K_D_⋅K_S_ = 6.265∙10^8^ M^−2^. Consequently, we assign K_S_ = 5.97∙10^4^ M^−1^ and K_D_ = 1.05 ⋅ 10^4^ M^−1^. Hence, the quenching mechanism of ChA on the luminescence of Tb-R_3_ complex is combined static and dynamic.

## Conclusions

We describe the luminescence properties of three new terbium complexes with 1-(furan-2-ylmethyl)-4-hydroxy- 2-oxo-1,2,5,6,7,8-hexahydroquinoline-3-carboxamide ligands. The complex of one of these ligands (R_3_) in 1:1 molar ratio with Tb^3+^ is a highly sensitive and selective probe for the determination of chlorogenic acid by luminescence quenching. This enables the reliable and accurate determination of ChA in urotropine buffer at near neutral pH as validated by a concomitant determination of ChA with HPLC. The ChA contents found by luminescence determination in real green and roasted coffee samples agreed very well with those of HPLC. Luminescence lifetime measurements show contributions of both static and dynamic quenching and the related quenching constants are K_S_ = 5.97∙10^4^ M^−1^ and K_D_ = 1.05⋅10^4^ M^−1^. The proposed method is simple, yet very accurate and reproducible and can be used for the quality control of ChA in coffee samples prior and after roasting.

## Supplementary Information

Below is the link to the electronic supplementary material.

Supplementary file1 (DOCX 30 KB)
