# Supplementary material for: New terbium complex as a luminescent probe for determination of chlorogenic acid in green coffee and roasted coffee infusions
Source: Anal Bioanal Chem. 2022 Nov 15;415(2):235–44. doi: 10.1007/s00216-022-04411-x (PMC9823076; doi:10.1007/s00216-022-04411-x)
Supplement: Supplementary file 1 — Supplementary file1 (DOCX 30 KB) [file 216_2022_4411_MOESM1_ESM.docx]

Supplementary information to

**New terbium complex as a luminescent probe for determination of chlorogenic acid in green coffee and roasted coffee infusions**

Alla Yegorova^1*^, Yuliia Skrypynets^1^, Inna Leonenko^1^, Axel Duerkop^*2^

^1^A. V. Bogatsky Physico-Chemical Institute, National Academy of Sciences of the Ukraine,

Lustdorfskaya doroga 86, Odessa 65080, Ukraine

^2^Institute of Analytical Chemistry, Chemo- and Biosensors, University of Regensburg, Universitätsstrasse 31, D-93040 Regensburg, Germany

**Fig. S1** Effect of organic solvents (50 % v/v) on the luminescence intensity of the Tb-R_3_ complex (c_Tb-R3_ = 1.00∙10^-5^ mol/L; λ_exc_ = 315 nm, λ_em_ = 545 nm): 1 – water (without organic solvent added), 2 - ethanol, 3 – methanol, 4 - isopropanol, 5 - dimethyl sulfoxide, 6 - dimethyl formamide
